# Fusing Accelerometry with Videography to Monitor the Effect of Fatigue on Punching Performance in Elite Boxers

**DOI:** 10.3390/s20205749

**Published:** 2020-10-10

**Authors:** Nicos Haralabidis, David John Saxby, Claudio Pizzolato, Laurie Needham, Dario Cazzola, Clare Minahan

**Affiliations:** 1 Department for Health, University of Bath, Bath BA2 7AY, UK; ln424@bath.ac.uk (L.N.); d.cazzola@bath.ac.uk (D.C.); 2 School of Allied Health Sciences, Griffith University, 4222 Gold Coast, Australia; d.saxby@griffith.edu.au (D.J.S.); c.pizzolato@griffith.edu.au (C.P.); c.minahan@griffith.edu.au (C.M.); 3 Griffith Centre of Biomedical and Rehabilitation Engineering, Menzies Health Institute Queensland, Griffith University, 4222 Gold Coast, Australia

**Keywords:** inertial measurement unit, optimal control, direct collocation, inverse dynamics, upper-limb kinetics

## Abstract

Wearable sensors and motion capture technology are accepted instruments to measure spatiotemporal variables during punching performance and to study the externally observable effects of fatigue. This study aimed to develop a computational framework enabling three-dimensional inverse dynamics analysis through the tracking of punching kinematics obtained from inertial measurement units and uniplanar videography. The framework was applied to six elite male boxers performing a boxing-specific punch fatigue protocol. OpenPose was used to label left side upper-limb landmarks from which sagittal plane kinematics were computed. Custom-made inertial measurement units were embedded into the boxing gloves, and three-dimensional punch accelerations were analyzed using statistical parametric mapping to evaluate the effects of both fatigue and laterality. Tracking simulations of a sub-set of left-handed punches were formulated as optimal control problems and converted to nonlinear programming problems for solution with a trapezoid collocation method. The laterality analysis revealed the dominant side fatigued more than the non-dominant, while tracking simulations revealed shoulder abduction and elevation moments increased across the fatigue protocol. In future, such advanced simulation and analysis could be performed in ecologically valid contexts, whereby multiple inertial measurement units and video cameras might be used to model a more complete set of dynamics.

## 1. Introduction

Successful performance in the sport of boxing requires the boxer to deliver impactful punches with both hands despite accumulating fatigue. Pierce et al. [1] demonstrated winning boxers deliver higher impact punches compared to their defeated opponents, stressing the importance of both punch force and velocity. Furthermore, higher ranked amateur boxers possess greater muscular strength and power, as well as higher aerobic and anaerobic power, compared to those lower ranked [2]. These fundamental physiological components of fitness can be developed through strength and conditioning training [3] and are critical for maintaining punch force and velocity during boxing competition [2]. Importantly, bilateral delivery of impactful punches depends, in part, on biomechanical coordination, which may be negatively affected by fatigue. Although it is presumed elite boxers have minimal bilateral punching asymmetry [4], it is unclear what effects prolonged punching might have on asymmetries.

Advances in technology has led to use of wearable sensors to address numerous clinical (e.g., aging and osteoarthritis [5]; sit-to-stand performance [6]) and sport (e.g., classifying training exercises [7] and running [8]) biomechanical research avenues. Within boxing, wearable sensors alongside motion capture technology are accepted instruments to measure spatiotemporal variables for quantitative description of punching performance, however, their combined use for such purposes has not been undertaken previously. Amongst the existing boxing literature, punch acceleration is well documented [9,10], and analysis confirms common knowledge that prolonged punching output (e.g., during training or competition) reduces punching speed. Punch impact forces have also been assessed, revealing elite boxers generate injurious impacts when applied to head-forms [11] and human opponents [1,12,13] alike. For training purposes, inertial measurement units (IMU) embedded within boxing gloves have been used to automatically detect fatigue by analyzing punch kinematics [10]. Although this approach may aid fitness assessment or monitoring training progression, it does not reveal underlying mechanisms for the decline in punch velocity due to fatigue.

Computational modeling of the neuromuscular system is one way to study causal mechanisms behind sporting movements, such as punching. However, robust computational modeling requires experimental measures of task kinematics and kinetics to constrain simulations to physiological solutions. In sport settings, athlete kinematics can be acquired through simple and inexpensive instruments (e.g., uniplanar videography), from which two-dimensional segment and joint motions can be calculated. Unfortunately, traditional videography processing is manual, tedious, time-consuming, and unreliable. Advances in computer vision and artificial intelligence provide alternatives to traditional videography processing [14]. Technologies, such as OpenPose [15,16], use deep convolutional neural networks to identify body landmarks from sparse imaging and have been shown to be accurate (e.g., 2–4 cm errors) for walking, running, and throwing [17]. Experimental kinematics obtained from combined use of wearable IMU and uniplanar videography have received little attention within the computational modeling literature. Combining wearable IMU and uniplanar videography with computational modeling will yield more extensive insight into task kinetics (i.e., net joint forces and moments) and neural controls associated with such movements. Importantly, knowledge of the causal mechanisms behind movement, such as punching within boxing, might inform technique-related coaching frameworks and strength and conditioning programs to develop specific motions, net joint moments, and their coordination to improve performance.

The primary aim of the current study was to develop a novel computational framework for biomechanical analysis of punching. The computational framework featured a three-dimensional inverse dynamics analysis performed on data simulated by tracking uniplanar videography and wearable sensor data. The second aim was to apply the framework to determine how a boxing-specific punch fatigue protocol affects upper-limb net joint moments generated during punching in elite level boxers and to explore the potential effects on punching laterality. We hypothesized that, in response to a boxing-specific punch fatigue protocol, punch accelerations would decline as would net shoulder and elbow moments. As very little is understood of the effects of a fatiguing protocol on punching biomechanics, we explored if laterality effects emerged across the punching protocol.

## 2. Materials and Methods

### 2.1. Participants

Participants were recruited from Pacific Island Nations involved in an athletics program in partnership with Griffith University in preparation for the 2018 Commonwealth Games. From the Gather Adjust Prepare Sustain (GAPS) program, six right-handed male elite boxers participated. They were (mean ± standard deviation) 1.68 ± 0.03 m in stature, 64.4 ± 4.6 kg in mass, 20 ± 2 years of age, and had 77.5 ± 4.4 cm of reach. Participants provided their informed written consent prior to testing taking place. The study was conducted in accordance with the Declaration of Helsinki, and the research protocol was approved by Griffith University’s human ethics research committee (ENG/14/13/HREC; AHS/2016/888/HREC). 

### 2.2. Sensors, Experimental Design and Data Acquisition

Overall, the experiment consisted of concurrent and synchronous use of IMU and uniplanar videography to acquire upper-limb kinematics during a boxing-specific punch fatigue protocol. The accelerometers within the IMU measured three-dimensional linear accelerations, which were stored on-board using micro secure digital (SD) cards and later transferred to a personal computer. Videography data were post-processed using a novel deep learning algorithm (i.e., OpenPose) to determine upper-limb segmented kinematics projected into the sagittal plane of motion. Together, these two sources of experimental kinematic data were used, along with a musculoskeletal model (in OpenSim), in an optimization framework to resolve generalized joint loads acting in three-dimensions.

To measure punching accelerations, each participant was outfitted with two IMU. The IMU were placed, one for left and right sides, atop the dorsal surface of the distal forearm, approximately half-way between radial and ulnar prominences and fixed using the strap of the punching glove. Relative to the anatomical position (i.e., quiet stance, elbow straight, forearms supinated, and arms by the sides), the IMU were orientated with x-axis (+) down, y-axis (+) towards radius, and z-axis as cross-product of x- onto y-axes. Due to the large accelerations that occur when a punch impacts a massive object (e.g., wall-mounted bag), custom built IMU were used (SABELSense, Griffith University, Nathan, Australia) (see for details [18,19]). These IMU were calibrated [20] off-line prior to testing, and data were logged to an on-board micro-SD card. The IMU, of dimensions 55 mm × 30 mm × 13 mm (length, width, and height, respectively), weighed approximately 23 g. The low-profile lightweight design minimized interference with normal punching conditions. Each IMU consisted of a ±7 Gauss 3-dimensional magnetometer, ± 4000 degree/s 3-dimensional gyroscope, and a ± 400 g 3-dimensional accelerometer. The IMU sampled at 250 Hz, which is considered adequate for measuring human punch impacts [21]. The IMU included a red light-emitting diode that was manually pulsed while in the video camera’s field of view to enable synchronization with videography data after testing was completed.

**Figure 1:**
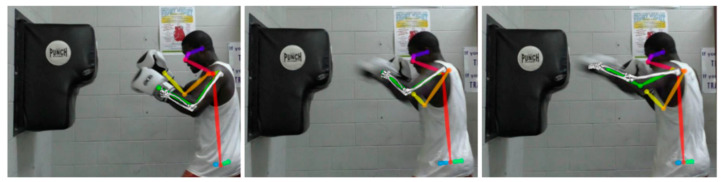
Exemplar of sequential frames from the video camera recording showing the upper-limb joint centers, estimated using OpenPose, and the left arm kinematic solution from the corresponding tracking simulation.

During the punch fatigue protocol, a standard video camera (HC-V750M, Panasonic, Osaka, Japan), sampling at 50 Hz, was positioned roughly halfway between the stance position of the participant and the wall-mounted punch bag (Figure 1). The camera was elevated to the approximate height of the punch path (estimated as participant shoulder height) and oriented such that the primary axis was perpendicular to the punch path, exposing the boxers’ left side to the camera. Given this setup, videography data were only available for left side punches.

The experiment began by affording each participant an unstructured 10 min warm-up, after which they performed a boxing-specific punch fatigue protocol using their own boxing gloves and IMU embedded underneath (explained above). The punch fatigue protocol involved 11 sets of 5-s maximal velocity punch bursts followed by 5-s of rest. Each punch burst started with a left punch delivered to the punch bag (Punch Equipment, Gold Coast, Australia), which was wall-mounted at a height of 1.7 m from the ground, followed by a succession of alternating left and right straight punches. Participants were instructed to remain with their feet in a fixed position (e.g., do not advance or change stance) and to punch as straight, fast, and hard as possible during each 5 s burst. Coaches and teammates provided vigorous verbal encouragement during the protocol, and it was evident from the facial expressions and utterances of the participants that the protocol elicited many uncomfortable sensations.

### 2.3. Signal Processing

Bursts and individual punches were identified from the series by using the anterior–posterior (AP) component of the IMU acceleration through a custom peak identification algorithm. The ‘*findpeaks*’ function in Matlab (version 7.5, 2016b, MathWorks Inc., Natick, MA, USA) was applied to the derivative of AP acceleration. From this derivative, the locations of the peaks were used to identify start and end of each burst. To identify the onset and end of each individual punch, peaks in the AP accelerations within each burst were found using a second derivative method on the normal and flipped versions of the signal.

The punch cycle comprised three phases: *pre-impact*, *impact* and *retraction*. The *pre-impact* phase was characterized by a positive acceleration towards the wall-mounted punch bag caused by arm extension. This phase started when AP acceleration was zero and ended at impact, which was identified by the acceleration crossing zero again (i.e., instant of maximum positive velocity) but before the glove impacted the wall-mounted punch bag (i.e., maximum negative AP acceleration). The *impact* phase was characterized by a negative acceleration and started at the end of time of impact and ended at the beginning of the *retraction* phase. The *retraction* phase started at the instant when the acceleration crossed zero after time of impact. To perform the biomechanical simulations, IMU and videography data from solely the *pre-impact* phase were used. For the fatigue and laterality analysis of the punch accelerations, IMU data from both the *pre-impact* and *impact* phases were considered.

### 2.4. OpenPose Methods

Marker-free estimation of upper-limb joint centers was achieved using OpenPose [15]. Each image from the video set was input to the OpenPose network, which returned a two-dimensional probability map of the location of the wrist, elbow, and shoulder joint centers. For each detection by OpenPose, the local maxima of the probability map were considered the joint center coordinates. To track each joint center through time and eliminate spurious tracking (due to multiple people within the camera’s field of view), the Euclidean distance between joint center detections in successive video frames was minimized. Finally, joint center locations were filtered using a fixed-interval Kalman smoother [22,23] to provide optimal state estimation of each joint center location over time.

### 2.5. Optimal Control–Direct Collocation

From all punches in the protocol, simulations for a sub-set of left-handed punches were formulated as optimal control problems and converted to nonlinear programming problems for solution with a trapezoid direct collocation method [24]. For each of these simulations, we used an adapted version of the three-dimensional upper-limb model (see Supplementary Material Figure S1) [25] distributed with OpenSim (version 3.3, Stanford University, Stanford, CA, USA) [26]. The upper-limb was modeled as a multibody system, comprised of 4 rigid segments (humerus, radius, ulna, and hand), and 5 degrees-of-freedom (shoulder flexion–extension, adduction–abduction, and internal–external rotation as well as elbow flexion–extension and pronation–supination).

The objective of these simulations was to determine model state and control variables that tracked experimental kinematic data (i.e., accelerations from IMU and videography-based segment angles), while satisfying both multibody dynamics and boundary constraints. State variables were the model’s generalized coordinates and velocities, while control variables were the model’s generalized accelerations. Using generalized accelerations as control variables avoided their calculation explicitly from the equations of motion, which can lead to numerical difficulties when inverting the model mass matrix [27]. In total, the model had 10 state and 5 control variables. For each simulation, we discretized state and control variables into 35 equally spaced nodes to enable the differential equations describing our multibody dynamic constraints to be replaced with algebraic equality constraints using the trapezoid method [24]. We also included inequality boundary constraints to ensure solutions were physiological.

For each simulation, we tracked accelerations measured by IMU as well as upper-arm and forearm segment angles calculated from the joint center coordinates estimated by OpenPose (Figure 1). To obtain equivalent simulated kinematics and net joint moments, we used methods within the OpenSim C++ application programming interface (API) (version 3.3). This was achieved by a Matlab executable (MEX) function that provided an interface between OpenSim C++ API and Matlab for a set of discretized state and control variables. For the simulations, we assumed the IMU was placed at the hand’s center of mass. The objective function for the simulations contained weighted terms to minimize the squared differences between experimental and simulated data, and to also minimize the squared generalized acceleration control variables:

(1) $$\begin{array}{c} J=w_{1}\overset{2}{\underset{j=1}{\sum }}\int_{t_{0}}^{t_{f}}{\left(\frac{q_{j}^{EXP}\left(t\right)-q_{j}^{SIM}\left(t\right)}{range\left(q_{j}^{EXP}\right)}\right)}^{2}dt+w_{1}\overset{3}{\underset{i=1}{\sum }}\int_{t_{0}}^{t_{f}}{\left(\frac{acc_{i}^{EXP}\left(t\right)-acc_{i}^{SIM}\left(t\right)}{range\left(acc_{i}^{EXP}\right)}\right)}^{2}dt\\ +w_{2}\overset{5}{\underset{k=1}{\sum }}\int_{t_{0}}^{t_{f}}{\left(\frac{u_{{\ddot{q}}_{k}}^{SIM}\left(t\right)}{bound\left(u_{{\ddot{q}}_{k}}^{SIM}\right)}\right)}^{2}dt \end{array}$$

where, superscripts *EXP* and *SIM* denote experimental and simulated data, respectively, $q_{j}$ and $acc_{i}$ represent the jth segment angle and ith accelerometer signal, respectively, $u_{{\ddot{q}}_{k}}$ corresponds to the kth generalized acceleration control variable, $t_{0}$ and $t_{f}$ denote initial and final times, respectively, of experimental data per punch cycle being tracked, and $w_{p}$ are weightings whose values were set based upon the importance of the term included in the objective function ($w=\left[5\;5\times 10^{-5}\right]$). We included minimization of control variables with a small weighting to avoid redundancy and improve convergence. Each of the tracked variables within the objective function were normalized by the range of experimental data from the tracked punch. Control variables were normalized by their permissible bound determined from experimental data.

In total, we performed 144 simulations (6 participants × 24 punches). For each participant, we tracked the 1st, 4th, 8th, and 12th punches from the 1st, 3rd, 5th, 7th, 9th, and 11th bursts of the punch fatigue protocol. Each simulation commenced at the minimum upper-arm segment angle and terminated at the frame prior to impact (as determined by the accelerometer signals within the IMU). The constrained minimization function, ‘fmincon’, in the Matlab optimization toolbox in combination with OpenSim were used to solve each nonlinear programming problem. We used an interior-point solver with the objective function and constraint tolerances set to ${1\times 10}^{-4}$ and ${1\times 10}^{-5}$, respectively.

### 2.6. Statistical Analysis

#### 2.6.1. Fatigue and Laterality Analysis on Punch Acceleration

A fatigue analysis was carried out comparing left and right punch acceleration components (i.e., AP, medial–lateral (ML), and cranial–caudal (CC)) and resultant from the ‘non-fatigued’ burst (i.e., first burst of the protocol from each participant) with the following 10 ‘fatigued’ bursts. The comparisons were performed using statistical parametric mapping (SPM) [28] and a within-subject analysis one-way repeated measure analysis of variance (ANOVA).

A laterality analysis was carried out comparing the acceleration components and resultant from right and left punches within each burst of the protocol (i.e., theoretically same fatigue level). The comparisons were performed using SPM and a two-way repeated measure ANOVA, where the main effects were fatigue and laterality, with their interaction modeled.

#### 2.6.2. Fatigue Analysis of Net Joint Moments

A fatigue analysis was carried out comparing simulated net moments from the left arm from the ‘non-fatigued’ burst (i.e., first punch of the first burst for each participant) with the corresponding simulated net joint moments of the 5 subsequent ‘fatigued’ bursts (i.e., bursts 3, 5, 7, 9 and 11). The joint moments were shoulder elevation, shoulder abduction/adduction, elbow flexion/extension, and elbow pronation/supination. Main effects were analyzed using SPM with a within-subject analysis one-way repeated measure ANOVA.

## 3. Results

### 3.1. Fatigue and Laterality Effects on Punch Acceleration

**Figure 2:**
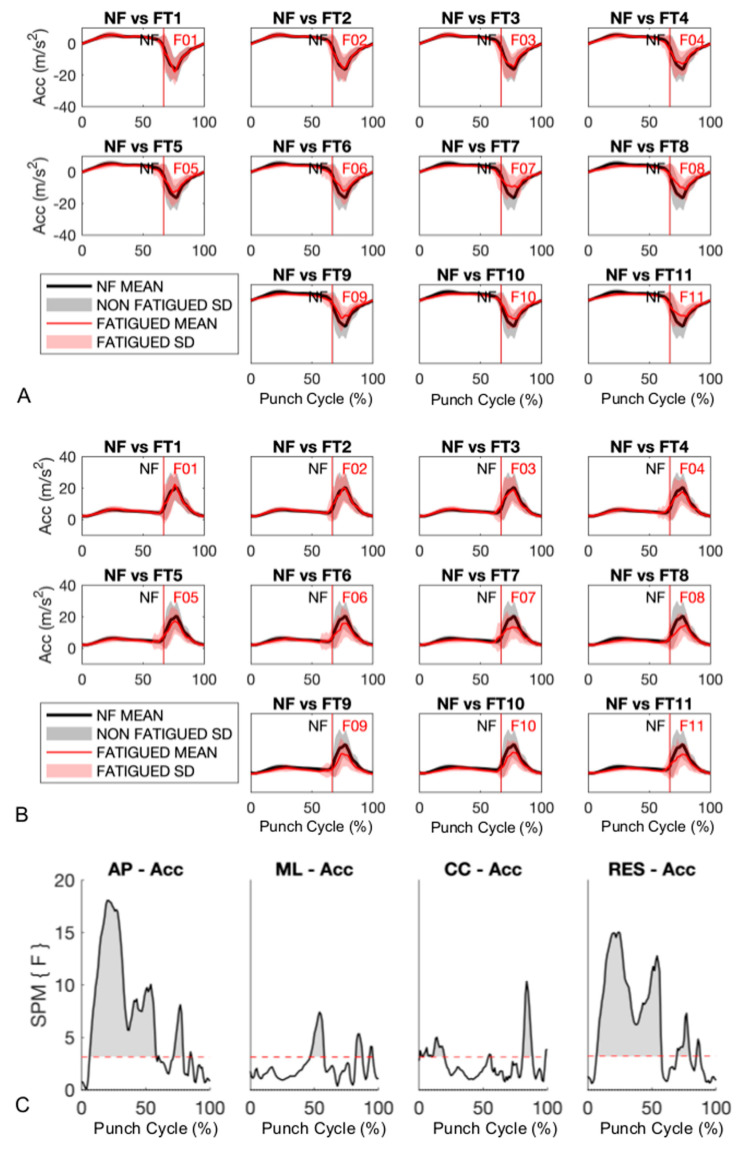
(**A**) Anterior–posterior (AP) component and (**B**) resultant accelerations. Vertical lines show average instant (% of cycle) of impact for non-fatigued (NF) and fatigued (F) trials, while 0% and 100% correspond to the beginning of the pre-impact and end of the impact phases, respectively. (**C**) Results from within-subject one-way ANOVA using a statistical parametric mapping approach (SPM{F}). ML—medial–lateral; CC—cranial–caudal; RES—resultant; Acc—acceleration.

A fatigue effect was evident in the AP acceleration component (Figure 2A), which is the predominant component during straight punching. Similarly, a decrease in resultant acceleration due to the punch protocol was observed (Figure 2B). The AP component of acceleration significantly decreased during pre-impact (6% to 58% (*p* < 0.001)) and impact (60% to 79% (*p* < 0.001)) phases (Figure 2C, grey shaded regions). These decreases in AP acceleration are visually apparent from the 6th punch burst onwards (Figure 2A,B). Moreover, from the 7th punch burst onwards, decreases in the slopes of acceleration (i.e., jerk) components are observed, likely due to decreased rate of force development as a function of fatigue. A similar decrease in resultant acceleration and jerk is visible and confirmed by statistical analysis (Figure 2).

As the punch protocol progressed, ML and CC components of acceleration (and jerk) showed significant decreases, but less consistently compared to the AP component. Decreases in accelerations (and jerk) were observed during both pre-impact (0% to 70% (*p* < 0.001) and impact (70% to 100% (*p* < 0.001)) phases for ML (46% to 57% (*p* < 0.001), 82% to 87% (*p* < 0.001), and 92% to 95% (*p* = 0.0071)) and CC (0.4% to 2.6% (*p* = 0.018), 3.1% to 9.7% (*p* < 0.001), 10.3% to 19.8% (*p* < 0.001), 54% to 56% (*p* = 0.0154), 80.7% to 88.5% (*p* < 0.001), and 98.6% to 100% (*p* = 0.0282)) components (Figure 2).

**Figure 3:**
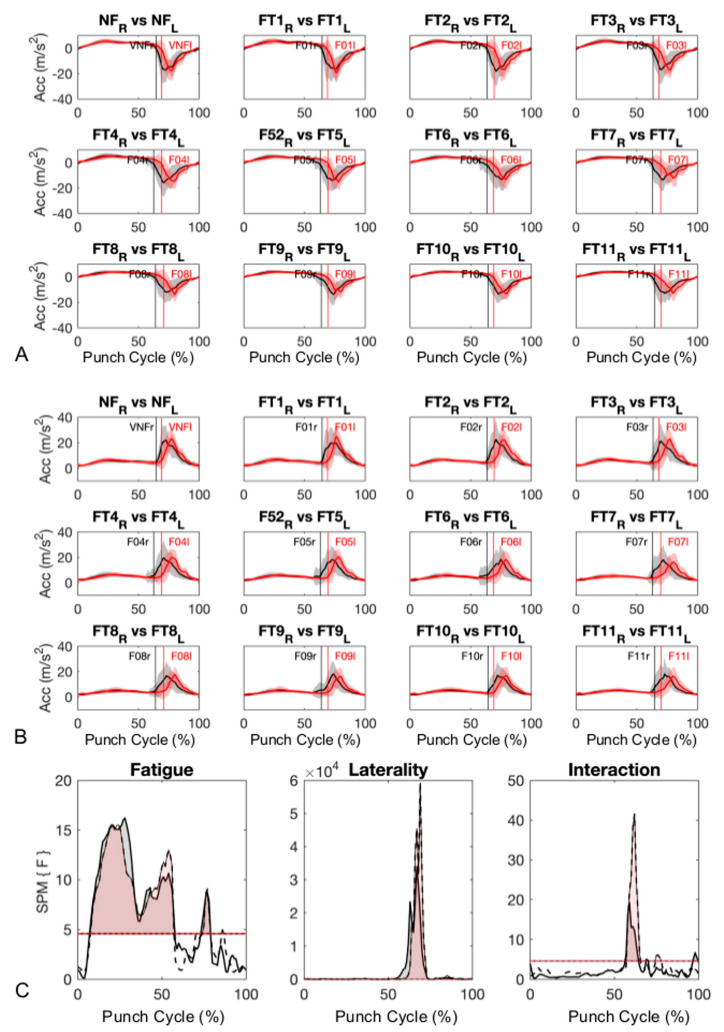
(**A**) Anterior–posterior (AP) and (**B**) resultant acceleration for right (black lines) and left (red lines) hand punches. Vertical lines show average impact point for both fatigued (FT) and non-fatigued (NF) trials, while 0% and 100% correspond to the beginning of the pre-impact and end of the impact phases, respectively. (**C**) Results from the within-subject one ANOVA using a statistical parametric mapping approach for AP component (solid black line) and resultant (dashed black line) accelerations. Periods showing a statistically significant difference are highlighted by grey (anterior–posterior) and red (resultant) areas above critical F statistic.

Using a two-way (i.e., fatigue, laterality, and interaction) ANOVA statistical parametric mapping, a significant main effect of fatigue was found during both pre-impact and impact phases for AP (6% to 59% (*p* < 0.001) and 71% to 79% (*p* < 0.001)) and resultant (6% to 58% (*p* < 0.001), 69% to 79% (*p* < 0.001) and 84% to 87% (*p* = 0.0019)) accelerations (Figure 3C). A significant effect of laterality (Figure 3C) in both AP (0% to 3% (*p* = 0.0012), 8% to 18% (*p* < 0.001), 29% to 40% (*p* < 0.001), 47% to 74% (*p* < 0.001), 77% to 92% (*p* < 0.001), 97% to 100% (*p* < 0.001)) and resultant (0% to 6% (*p* = 0.0012), 10% to 21% (*p* < 0.001), 26% to 44% (*p* < 0.001), 57% to 74% (*p* < 0.001), 76% to 94% (*p* < 0.001), and 99% to 100% (*p* < 0.001)) accelerations was also found. Finally, a significant interaction effect between fatigue and laterality was found for AP (0% to 0.3% (*p* = 0.048), 55% to 65% (*p* < 0.001), 68% to 70% (*p* < 0.012), and 95% to 100% (*p* < 0.001)) and resultant (57% to 66% (*p* < 0.001), 68% to 70% (*p* < 0.019), 73% to 78% (*p* = 0.019), and 82% to 83% (*p* = 0.049)) accelerations. From inspection of the AP acceleration component (Figure 3A), it was evident the impact from right-hand punches occurred earlier compared to left-hand punches, independent of fatigue state. This trend was also mirrored in the resultant accelerations (Figure 3B) and confirms a laterality effect (Figure 3C) from the instant of impact to the beginning of the retraction phase.

### 3.2. Data-Tracking Simulations

**Figure 4:**
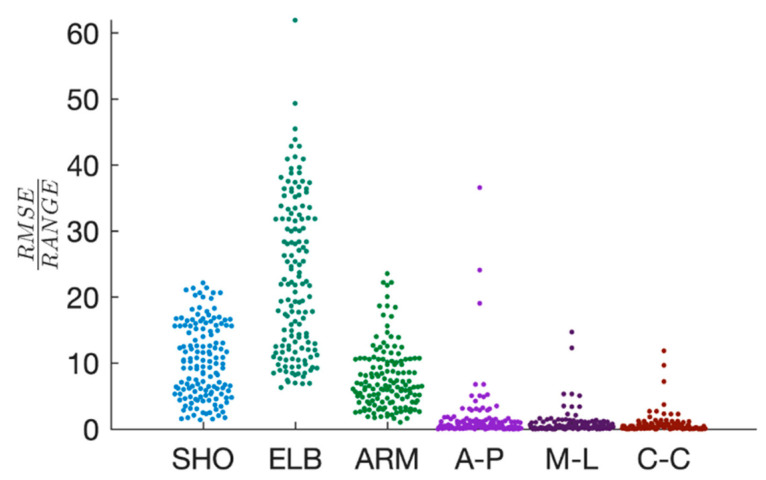
Distribution plots showing tracking performance reported as root mean squared error (RMSE) expressed as percentage of maximal range of motion or acceleration. Shoulder (SHO) and forearm (ARM) angles were directly tracked within the optimization, as well as anterior–posterior (AP), medial–lateral (ML) and cranial–caudal (CC) hand acceleration components. Elbow joint angle (ELB) error is also shown, although this angle was not directly tracked within the optimization.

Segment angles from the upper-arm and forearm were tracked with average error <10% of the segment’s angular range of motion calculated from videography data through OpenPose. Punch accelerations were tracked with average error <2% of the acceleration range. Shoulder elevation was tracked with average error ~10%, whereas the forearm segment angle was tracked with average error ~7.6% (Figure 4). Wrist AP, ML, and CC acceleration components were tracked with average errors, normalized to experimental range, of 1.5%, 0.8%, and 0.6%, respectively (Figure 4).

**Figure 5:**
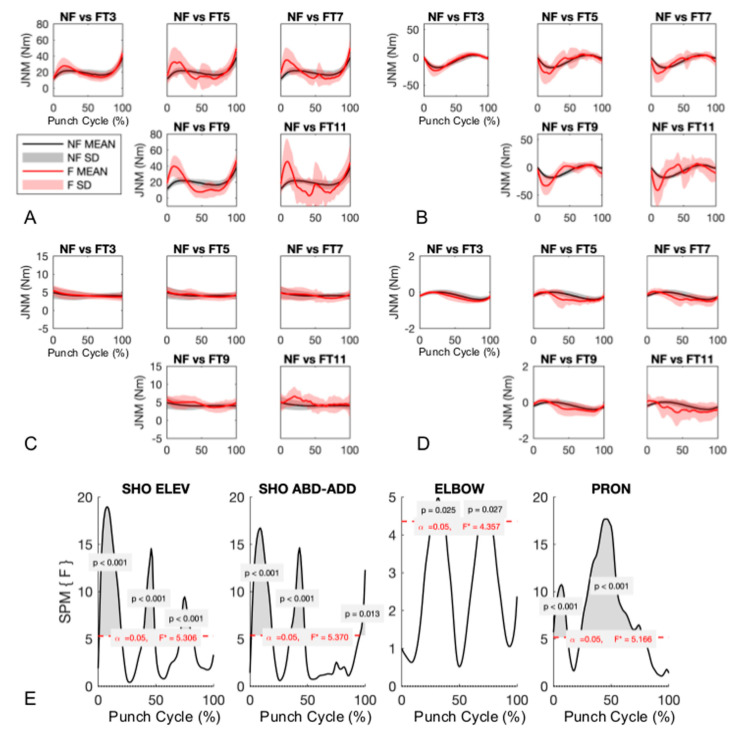
(**A**) Shoulder elevation, (**B**) shoulder abduction/adduction, (**C**) elbow flexion/extension, and (**D**) elbow pronation/supination moments. Non-fatigued (NF) (black solid lines and bands) plotted against 5 different fatigued (FTX) trials (red lines and bands). (**E**) Results from within-subject one-way ANOVA using a statistical parametric mapping approach. Periods showing a statistically significant difference are highlighted by grey areas above the critical F statistic. The simulations were carried out from the beginning of the pre-impact phase (0%) to the end of the pre-impact phase (100%).

Net shoulder and elbow moments during non-fatigued and fatigued punch bursts were similar for initial and final portions of pre-impact (Figure 5A and B). Across the five bursts, a main effect of fatigue on joint moments was found (Figure 5E). Specifically, shoulder elevation moment significantly increased (0.7% to 20%, (*p* < 0.001)), followed by a decrease towards the middle and final portions of pre-impact phase (38% to 49% (*p* < 0.001), 71% to 81% (*p* < 0.001)). The shoulder abduction/adduction moment similarly increased (1% to 19% (*p* < 0.001)) and then decreased (34% to 47% (*p* < 0.001), 95% to 100% (*p* = 0.013)) in magnitude. The elbow flexion/extension moment slightly increased (26% to 37% (*p* = 0.025), 67% to 77% (*p* = 0.027)) during pre-impact phase. Finally, elbow pronation/supination moment slightly increased (0% to 12% (*p* < 0.001), 23% to 77% (*p* < 0.001) during pre-impact phase.

## 4. Discussion

This study integrated open-source pose estimation software, biomechanical modeling, and an optimal control approach to understand performance of elite boxers during a boxing-specific punch fatigue protocol. As the boxers progressed through the protocol, their net shoulder moments changed significantly, first increasing in magnitude and then decreasing, whereas the elbow experienced small load modulations. Moreover, we found an interaction between laterality (i.e., left and right punches) and decreased punch accelerations as the protocol progressed. Specifically, the boxers’ habitual lead hand (i.e., front hand used for jabbing) did not lose acceleration as quickly as their rear hand (i.e., crossing hand). Importantly, the joint moments could not have been deduced from inspection of the acceleration signals or videography data, but required an integrated simulation framework that accurately tracked kinematics (i.e., segment angles and IMU accelerations) while enabling analysis of punch kinetics. In future, such advanced simulation and analysis could be performed in ecologically valid contexts, whereby multiple IMU and video cameras might be used to model a more complete set of dynamics.

A key to extracting value from wearable sensor data for sport, medical, and industrial applications is to ensure sensor measurements can be associated with meaningful physical processes (e.g., body accelerations or joint loads). Indeed, coaches, sports scientists, and clinicians can readily target body accelerations and joint loading through training and feedback. Physics-based models provide the critical mechanistic link between wearable sensor data and physical processes, however, these physics-based model must accurately predict physical phenomena: “a model is only as good as the data it can predict” (Professor David G Lloyd, personal communication, *saepe occurrentes*). It is important to acknowledge that even if a model well tracks measured punch (i.e., endpoint) accelerations, there still exists an infinite set of upper-limb kinematics that could produce these accelerations due to the redundant mobilities in the model (and the real human). However, our model tracked both three-dimensional punch accelerations and planar projections of the boxers’ upper-limb kinematics with little error, thus providing confidence our subsequent kinetic simulations were worth considering. Tracking both experimentally acquired endpoint accelerations and upper-limb kinematics was essential to establishing this framework as robust.

As hypothesized, the boxing-specific punch fatigue protocol resulted in a decrease in punch acceleration as a function of time, confirming common knowledge among athletes, coaches, and spectators alike. The decrease in punch acceleration was mainly evident in the AP component, but also found in the resultant. The predominance of the AP component within the resultant acceleration is unsurprising as the boxers were instructed to deliver straight punches throughout the protocol. As they were elite competitors, they executed our request faithfully as indicated by the minor contributions from ML and CC acceleration components to the resultant. As the punching protocol progressed, the SPM analysis highlighted a decrease in punch acceleration during the pre-impact phase (Figure 2), which indicated the boxers struggled to generate accelerations of the magnitude they produced at the commencement of the protocol. Likewise, the impact phase was affected by fatigue, but likely due to lower accelerations generated immediately prior to impact.

Although acceleration data from wearable sensors might be useful for analyzing punching performance, externally measured phenomena (e.g., accelerations) do not, by themselves, provide causal explanations of performance outcomes. Our presented framework enabled an analysis of the joint-level control of the upper-limb during the punch fatigue protocol, which revealed a clear change in the coordination of punching mechanics when the boxers approached their final bursts. Compared to the first burst of punches (i.e., non-fatigued), when fatigued the boxers generated greater shoulder abduction and elevation moments during the initial part of the pre-impact phase, but subsequently lower values at the instant of impact. A causal relationship exists between joint controls and punch accelerations, meaning fatigue affected shoulder moment generation, which is a key determinant of punching performance when considering the proximal-to-distal force generation pattern.

Our analysis revealed an interaction between fatigue (i.e., progression through the punch fatigue protocol) and laterality (i.e., dominant and non-dominant side) on punch accelerations, which was not anticipated. This interaction effect meant the loss of punch acceleration across the protocol was not equal between sides. This was particularly evident towards the end of the pre-impact and during impact phases (Figure 3), but in order to further explore this interaction a simple effect test must be performed. From visual inspection, the dominant hand fatigued faster, but we could not investigate this finding at the level of joint loading with our physics-based simulations as we were limited by having videography-based kinematics from only one side of the body due to the single video camera. Future work should consider bi-lateral videography if the goal is to elucidate laterality’s interaction with fatigue on the joint moments during punching.

The key requirements to enable coaches and training personnel to carry out biomechanical analysis are instrument affordability, ease in donning and doffing instruments, and speed/automation/insight of data analysis. Our framework was designed to be used with non-invasive standard equipment (i.e., one video camera, minimal IMU embedded within the gloves). Minimal equipment requirements are fundamental to ensuring computational technology, such as that implemented in the current study, can be adopted by coaches within ecologically valid training settings. However, such technology must still appropriately quantify performance biomechanics to provide actionable information for implementation. To this end, the simulation framework we present provides a means of generating dynamically consistent motions by integrating sagittal plane kinematics with 3D punch accelerometry. This was enabled by a multibody biomechanical model within a tracking optimization, which provided the anatomical and physical constraints to the optimizer to expand from simple videography and accelerometry to three-dimensional inverse dynamics analyses of punching kinetics. In the future, the net joint moments can be used to assess the punching strategy deployed by a boxer throughout a training session, and flag potentially inefficient or hazardous punching techniques to a coach for correction. If a physiologically plausible muscle-driven simulation could be generated for punching mechanics, as is regularly achieved for the lower-limbs [29,30] and sometimes achieved for the upper-limbs [31,32], a perturbation analysis [33] could also be performed to understand how muscles coordinate to accelerate the endpoint mass (hand).

The novel integration of a biomechanical model for punch analysis was useful to facilitate a functional interpretation of punch accelerations. Simultaneous tracking of video-based kinematics and wearable acceleration data in an optimal control problem was a novel approach, and not yet thoroughly applied in sport biomechanics, although elegant computational models for optimal shoulder control exist [27] as do methods to track IMU for determining lower-limb dynamics [8,34]. The biomechanical model allowed us to combined upper-limb joint kinematics with punch accelerations in a dynamically consistent manner, while ensuring the simulated kinematics matched the measured glove accelerations and segment angles. The use of a biomechanical model might be crucial in the future to providing feedback to coaches and athletes through model visualization solutions, as interpreting acceleration graphs can be very challenging and counterintuitive also for experts in the field.

Another important feature of the framework is the integration of marker-free estimation of body pose (OpenPose, [15]). One main challenge in sport biomechanics is to provide timely feedback to coaches and athletes. Therefore, algorithms, such as OpenPose that minimize manual digitization, are valuable as they reduce burden on users. Furthermore, OpenPose can generate segment (and joint) angles subsequently used within an optimization to determine joint controls. Such a marker-free motion capture approach might be scaled up for use with calibrated multi-camera systems to provide three-dimensional joint center estimates, which would be necessary for analysis of punches that occur across all three planes of motion (e.g., hooks) or for bilateral analysis.

### Limitations

There are limitations associated with this study that should be considered. First, this study did not use a gold standard method to determine upper-limb punch kinematics (i.e., three-dimensional optical motion capture systems). However, OpenPose has been found to predict joint center locations with errors of less than 20 mm in throwing tasks [17]. Despite excellent accuracy of the projected segment angles and three-dimensional punch accelerations, only the accelerations were directly tracked in the simulations, while the remaining model kinematics (e.g., shoulder abduction/adduction) were generated by the optimizer but subject to imposed constraints. In the future, multi-camera systems and/or IMU-based angular measures could be implemented to directly determine three-dimensional kinematics without compromising ecological validity. Second, the biomechanical model used for the physics-based simulations was a generic torque-driven model. The use of subject-specific models [35] has been shown to provide better results due to better representation of segment mass and inertia. However, this might not play a crucial role in this study as the trunk segment was fixed to the origin frame and simulations were performed during the pre-impact phase. Third, this study cannot provide insights into the neuromuscular strategies underpinning the changes in the boxing technique resulting from the punch protocol. To reveal the neuromuscular mechanisms, a muscle-driven simulation is needed and electromyographic measurements are recommended to enable neurally-informed solutions, as they have been shown to lead to more physiologically plausible results [36]. Fourth, the simulation framework was applied to the pre-impact phase of the punches. This was a necessary as it was not possible to create and validate a contact model due to the wall-mounted punch bag lacking instrumentation (i.e., pressure sensors), nor was it possible to quantify punch bag deformation during impact. Future studies may wish to pursue the creation and validation of such contact models to permit study of impact phase. Lastly, the cohort of boxers analyzed within this study were sampled conveniently and spanned three male weight classes according to the Olympic boxing federation. The weight classes are in place due to a heavy boxer being able to impart a more forceful punch than a lighter boxer, making for an unfair fight. However, it is possible the boxer from different weight categories may use different kinematic and kinetic patterns as they progress through a punch fatigue protocol. Our results must therefore be interpreted with some caution, as our study does not permit an analysis of the potential differences between weight classes in terms of their punch biomechanics throughout a fatigue protocol.

## 5. Conclusions

The framework presented in this study can be readily extended to other real-world scenarios to enable physics-based analysis of sport performance. In this study, we present a proof-of-concept application of our framework by studying the mechanistic effects of fatigue on boxing straight punching technique. This included analysis using SPM of both the experimentally measured punch accelerations and simulated upper-limb net joint moments across the fatiguing protocol. The results revealed the dominant limb fatigues more than the non-dominant, and the shoulder compensates with increased abduction and elevation moments.

## Supplementary Materials

The following are available online at https://www.mdpi.com/1424-8220/20/20/5749/s1.
