# Supplementary material for: Fusing Accelerometry with Videography to Monitor the Effect of Fatigue on Punching Performance in Elite Boxers
Source: Sensors (Basel). 2020 Oct 10;20(20):5749. doi: 10.3390/s20205749 (PMC7601017; doi:10.3390/s20205749)
Supplement: Supplementary file 1 [file sensors-20-05749-s001.pdf]

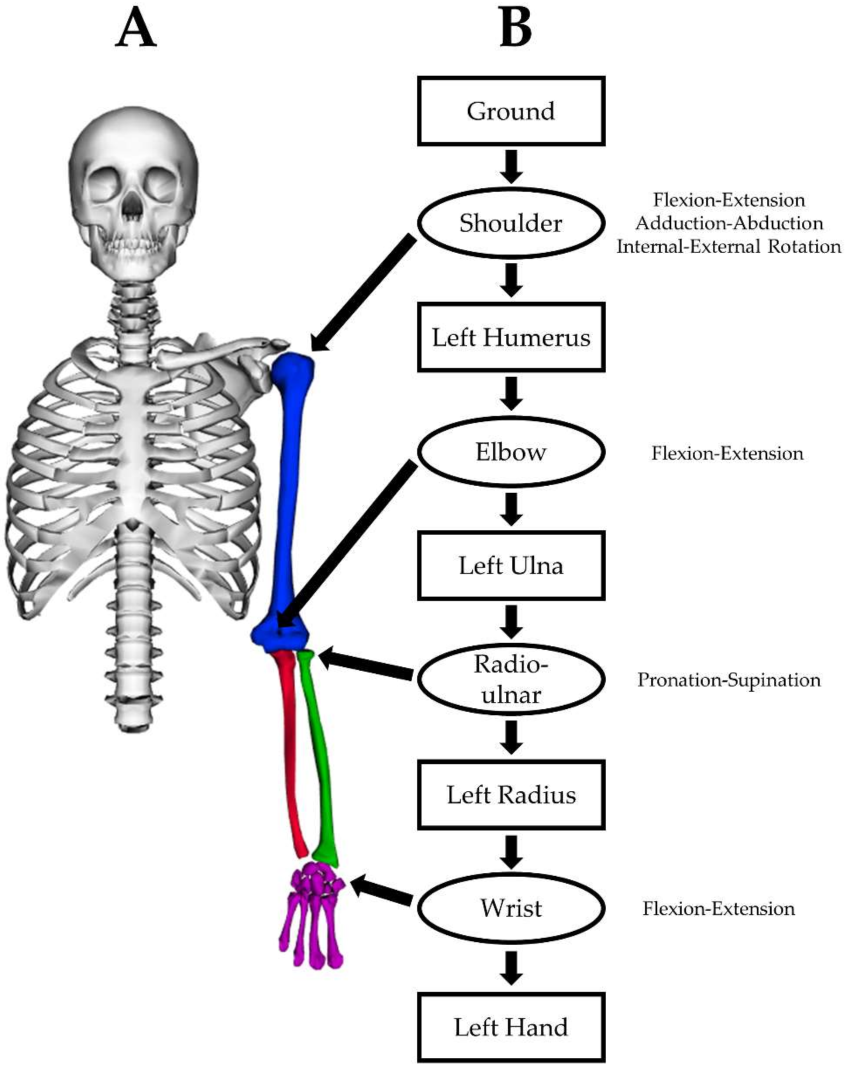

Supplementary Figure S1. (A) Image of three-dimensional model (torso and head geometry is purely for visualization purposes). (B) Model topology (rectangles: rigid segments; ellipses: joints with permissible degrees-of-freedom).
